# Influence of Brown Rice, Pea, and Soy Proteins on the Physicochemical Properties and Sensory Acceptance of Dairy‐Free Frozen Dessert

**DOI:** 10.1002/fsn3.4494

**Published:** 2024-10-09

**Authors:** Towhid Hasan, Yin Yin Thoo, Lee Fong Siow

**Affiliations:** ^1^ School of Science Monash University Malaysia Bandar Sunway Selangor Malaysia; ^2^ Department of Food Technology and Nutrition Science Noakhali Science and Technology University Noakhali Bangladesh

**Keywords:** brown rice protein, dairy‐free, frozen dessert, milk protein, pea protein, soy protein

## Abstract

Fat and protein derived from milk are prime ingredients in a frozen dessert such as ice cream conferring multiple desirable functionalities. However, this frozen dairy dessert is not suitable for individuals having lactose intolerance, cow milk allergy, or vegans. Hence, the study aimed to formulate dairy‐free frozen desserts using plant oils and plant proteins and compare their physicochemical characteristics and sensory acceptance against an ice cream containing milk fat and milk protein. Results indicated that the types of protein significantly influenced the physicochemical properties and sensory acceptance of the frozen dessert samples. Frozen desserts containing brown rice, pea, and soy protein showed greater resistance to melting (0.29, 0.12, and 0.19%/min vs. 1.95%/min), but they scored lower in sensory quality than ice cream made with milk protein; although they remained at an acceptable level. When compared among the plant proteins, the physicochemical characteristics of frozen desserts containing brown rice, pea, and soy protein varied because of the differences in the respective protein composition. Frozen dessert with brown rice protein showed higher overrun (47.50% vs. 40.78% and 37.8%), lower hardness (20.02 N vs. 22.24 and 26.37 N), and higher melting rate (0.29%/min vs. 0.19 and 0.12%/min) than frozen desserts containing soy and pea protein. Additionally, the brown rice protein frozen dessert received lower sensory acceptance than soy and pea protein frozen desserts. In summary, brown rice, pea, and soy proteins showed potential to be used as viable alternatives to milk protein for dairy‐free frozen dessert applications.

## Graphical abstract

In this study, dairy‐free frozen desserts were formulated from plant oils and plant proteins and compared against an ice cream containing dairy ingredients. The dairy‐free frozen desserts showed greater melting resistance than the dairy ice cream with acceptable sensory quality.

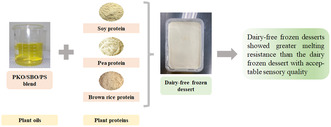

## 1 Introduction

Frozen desserts include a variety of foods such as ice cream, mellorine, sorbet, sherbet, frozen custard, frozen yogurt, and water ice that are commonly aerated and tested frozen (Goff and Hartel 2013; El‐Maksoud, Hesarinejad, and Abedelmaksoud 2023; Hasan, Thoo, and Siow 2023a). Among them, ice cream is the most consumed and famous frozen dessert. Ice cream is a multiphase food emulsion containing air cells, ice crystals, protein‐hydrocolloid structures, and partially coalesced fat globules dispersed in a continuous unfrozen aqueous matrix (Goff 1997a; Ghaderi, Mazaheri Tehrani, and Hesarinejad 2021). In general, it contains a mixture of sugar, stabilizer, fat, emulsifiers, solids‐non‐fat (SNF), and colors and flavors (Goff and Hartel 2013; Deosarkar et al. 2015; Shadordizadeh, Mahdian, and Hesarinejad 2023). Each component is added in different ratios within permissible limits to formulate ice cream. Due to specific regulation by the FDA (CFR 2019) regarding ingredient use, the term “frozen dessert” is used to describe the final product instead of ice cream.

Typically, a frozen dessert like ice cream contains 10%–12% fat, and fat is essential for many properties, including optimal structure, stability, and physical and sensorial attributes (Goff and Hartel 2013; Javidi and Razavi 2018; Mostafavi 2019). Milk fat such as anhydrous milk fats, cream, and butter serves as the major source of fat in frozen desserts (i.e., ice cream). The unique fatty acid profile of milk fat contributes to the characteristic flavor and provides a wide melting temperature (from −40°C to 40°C) (Ten Grotenhuis et al. 1999). These properties give rise to mouth‐coating characteristics and hence, an increased sensation of creaminess in the mouth (Goff 2006; Clarke 2012).

The SNF component of frozen desserts (i.e., ice cream) is responsible for the body and smooth texture, and proteins are a crucial member of the SNF. About 9%–12% SNF in a standard frozen dessert provides about 3%–4% protein (Goff and Hartel 2013). Milk proteins are preferred as the SNF source in frozen desserts formulation, primarily due to their contributions such as foam development and stabilizing the air cells, interacting with emulsifiers, stabilizing the fat globules, and enhancing the phase viscosity in addition to providing nutritional benefits (Goff and Hartel 2013; Chen et al. 2019; Shahi, Didar, et al. 2021; Shahi, Hesarinejad, et al. 2021).

Despite considerable benefits, frozen dairy desserts are not suitable for individuals having lactose intolerance and cow milk allergy, and who are vegan (Goff and Hartel 2013). Additionally, milk fat is more expensive than other edible fats/oils (Hasan et al. 2022). Consequently, food industries are looking for suitable substitutes to milk fat and milk proteins for frozen desserts (i.e., ice cream) formulation. In this regard, plant sources (i.e., plant oils and proteins) are getting attention as possible alternatives for dairy ingredients in the food products like frozen desserts (Ainis, Ersch, and Ipsen 2018).

Preparation of frozen desserts using plant oils is challenging since they have different techno‐functional properties than milk fat. A specific ratio of solid to liquid fat should be maintained to promote fat droplets to coalesce partially during the whipping/freezing process. This will help to constitute a network of fat globules building up the desired aggregated structure giving structural integrity to the frozen dessert (i.e., ice cream) (Goff 2006; Goff and Hartel 2013). Milk fat has the appropriate melting profile for making good frozen desserts because milk fat is predominantly but not completely solid at the aging temperature (4°C–5°C), where the fat structure is formed (Goff and Hartel 2013). In our earlier study (Hasan et al. 2022), we formulated ternary blends using palm kernel oil (PKO), soybean oil (SBO), and palm stearin (PS) that demonstrated a similar microstructure and solid fat content (SFC) profile as the milk fat. Hence, we concluded that these blends could be used as an alternative to milk fat for frozen dessert (i.e., ice cream) formulation.

Plant proteins like soy protein and pea protein confer certain benefits such as availability, acceptable cost, emulsifying properties, and high nutritive quality, making them a potential alternative to milk protein in food systems (Abdullah et al. 2003; Chen et al. 2019). However, it is vital to identify plant protein sources that imply similar physical and chemical characteristics compared to milk proteins for frozen dessert (i.e., ice cream) preparation.

Previous research demonstrated that the structural and quality characteristics of frozen desserts (i.e., ice cream) are greatly attributed to their emulsion or mix. On the other hand, the types of protein used play a crucial role in controlling the properties of food emulsions (Sourdet, Relkin, and César 2003; Relkin and Sourdet 2005). In our earlier work (Hasan, Thoo, and Siow 2023b), the effect of brown rice, pea, and soy protein on the frozen dessert mixes was assessed and compared against the milk protein. Better creaming stability, higher mix viscosity, and crystalline fat content were observed in the frozen dessert mixes that contained brown rice, pea, and soy protein compared to the frozen dessert mix prepared with the milk protein. From the findings, it was concluded that brown rice, pea, and soy protein possess the potential to be used in dairy‐free frozen desserts application as a substitute for milk protein.

Most of the preliminary studies investigated the effect of dairy ingredients (i.e., milk fat and milk protein) on frozen desserts such as ice cream (Goff, Kinsella, and Jordan 1989; Pelan et al. 1997; Alvarez et al. 2005; Patel, Baer, and Acharya 2006; Cheng et al. 2015; Daw and Hartel 2015). However, limited research has been conducted using soy protein (Dervisoglu, Yazici, and Aydemir 2005; Akesowan 2009; Cheng et al. 2016) and pea protein (Guler‐Akin, Avkan, and Akin 2021), and no study has been done applying brown rice protein for frozen desserts preparation. Additionally, little attention has been paid to preparing plant oil‐based frozen desserts using plant proteins as sources of solid non‐fat. Hence, the present research aimed to assess the physicochemical properties and sensory acceptance of frozen desserts (i.e., ice cream) containing dairy‐free ingredients (i.e., plant oil and proteins) and compare against an ice cream containing dairy ingredients (i.e., milk fat and milk protein).

## 2 Materials and Methods

### 2.1 Materials

Sugar, unsalted cow milk butter (Anchor, New Zealand), soybean oil (SBO), skim milk powder (Sunlac, New Zealand), and glyceryl monostearate (GMS) were purchased from a local supermarket at Bandar Sunway, Selangor, Malaysia. Palm stearin (PS) (iodine value: 38) and palm kernel oil (PKO) were obtained from Sime Darby Plantation, Malaysia. BLANOSE Cellulose Gum (CMC) was obtained from Markaids, Malaysia. Brown rice protein (78% protein), soy protein isolate, and pea protein isolate containing 90% and 80% protein, respectively, were purchased from Myprotein, Malaysia. The other chemicals were analytical grade and purchased from Sigma‐Aldrich, USA.

### 2.2 Preparation of Milk Fat and Oil Blend

Milk fat (> 99.9% fat) was prepared following our earlier study (Hasan et al. 2022). It was also observed in our earlier study that the ternary mixture of PKO/SBO/PS at ratios of 80/15/5 to 80/5/15 exhibited a similar solid fat content (SFC) like milk fat. Therefore, in this study, PKO/SBO/PS (80/15/5) was chosen as an alternative to milk fat.

### 2.3 Formulation of Frozen Dessert

The frozen desserts were formulated in accordance with Table 1. This formulation was commonly used in frozen dessert (i.e., ice cream) preparation (Goff and Hartel 2013). Milk fat was used as a fat source for the preparation of frozen desserts containing milk protein, while oil blend was used for the frozen desserts with brown rice, peas, and soy proteins. All the ingredients, excluding milk fat or oil blend, were mixed together followed by dispersing in warm water (50°C–55°C). The fats were melted and blended with the mixture under continuous stirring (approximately 15 min) to prepare premixes. The above premixes were pasteurized (65°C for 30 min) and subsequently mixed using IKA T25 digital Ultra‐Turax (Staufen, Germany) at 10,000 rpm for 2 min to make coarse emulsions. These emulsions were then homogenized using a twin‐stage valve homogenizer PandaPlus 2000 (Niro Soavi S.P.A., GEA, Italy) at 175 and 30 bar pressures at the first and the second stage, respectively. After that, the prepared mixes were kept at 4°C for overnight. Up to this stage, the sample was called frozen dessert mix. Prior to freezing, 0.2% vanilla extract was added to the mix. Then, the mixes were whipped and frozen in a smart scoop ice cream maker (BCI600; Breville, Australia) for 50 min. The frozen desserts were hardened at −18°C and stored at that temperature for 48 h before further analysis. The sample was termed frozen dessert after undergoing freezing.

**TABLE 1:** Formulations of frozen dessert (w/w%).

Ingredient (w/w%)	Frozen dessert			
	Milk protein	Soy protein	Pea protein	Brown rice protein
Fat	10	10	10	10
Milk protein	3.6	0	0	0
Soy protein	0	3.6	0	0
Pea protein	0	0	3.6	0
Brown rice protein	0	0	0	3.6
Sugar	15	15	15	15
Stabilizer	0.25	0.25	0.25	0.25
Emulsifier	0.1	0.1	0.1	0.1

### 2.4 Determination of Physicochemical Characteristics

The total solids (TS), fat, and protein content of melted frozen dessert samples were analyzed following the AOAC Official Method 941.08, 2000.18, and 991.20, respectively (AOAC 2012) with modifications. Total soluble solids (TSS, expressed in °Brix) were assessed using a pocket refractometer (PAL‐3; Atago Instruments, Japan). The pH was measured using a compact pH meter (LAQUAtwin pH‐33; Horiba Co., Japan) previously calibrated with pH 4.0 and 7.0 buffer solutions. The titratable acidity (TA) was determined according to AOAC Official Method 947.05 (AOAC 2012) with slight modifications.

### 2.5 Measurement of Color

The instrumental color of the frozen dessert samples was observed using a colorimeter (ColorFlex EZ; Hunterlab, Reston, VA, USA). The color attributes of lightness (L*), redness (a*), and yellowness (b*) were recorded using an illuminant D65 and 10° observer. The chroma (C*) and whiteness index (WI) of the samples were then calculated as below (Falah et al. 2021):

$$C*=\sqrt{a{*}^{2}+\;b\;{*}^{2}}$$

$$\mathrm{WI}=100-\sqrt{{\left(100-L*\right)}^{2}+a{*}^{2}+\;b{*}^{2}\;}$$

### 2.6 Analysis of Particle Size Distribution and Fat Destabilization

Laser dynamic scattering was employed to analyze the particle size distribution of frozen dessert mixes and melted frozen dessert samples using a Malvern Mastersizer 3000 system (Malvern Instruments Ltd., Worcestershire, UK) equipped with a Hydro EV liquid sample dispersion unit (Malvern Instruments Ltd.). The refractive indexes were 1.47 and 1.33 for dispersed and continuous phase, respectively, and the obscuration value was kept between 10% and 14%. Distilled water or 1% (w/v) sodium dodecyl sulfate (SDS) solution was used as diluents at 1:500 (w/v) ratio, and samples were analyzed at ambient temperature. The volume‐weighted average diameter (*d*_4,3_) was recorded and presented as the droplet size measurement. Partial coalescence degree (PCD), an irreversible aggregation of fat globules, was calculated using the following equation:

$$\mathrm{PCD}\;\left(\%\right)=\left[\left(d_{4,3\;\left(\mathrm{SDS}\right)}\;\text{of frozen dessert}\right)/\left(d_{4,3\;\left(\mathrm{SDS}\right)}\;\text{of frozen dessert}\;\mathrm{mix}\right)-1\right]\times 100$$

### 2.7 Determination of Desorption Index

Desorption index that usually characterize the extent of desorption of frozen dessert was measured following the methodology of Meneses et al. (2020) with slight modifications. Each 10 mL glass tube (height: 140 mm, inner diameter: 12 mm) was filled with melted frozen dessert samples, and stored for 1 h at ambient temperature. The tubes were sealed with parafilm before assessment. Desorption index (DI) was calculated by the following equation:

$$\mathrm{DI}\;\left(\%\right)=\left(H_{s}/H_{t}\right)\times 100$$

where *H*_s_ is the volume of the serum fraction and *H*_t_ is the total volume of the melted ice cream.

### 2.8 Analysis of Solvent Extractable Fat

Partial extraction of fat from frozen dessert samples was performed according to the methodology of Bolliger, Goff, and Tharp (2000) with slight modifications. About 10 g of melted frozen desserts were taken in a 50 mL centrifuge tube, and 30 mL of heptane was added. The mixture was slowly agitated for a few minutes, and the tube was kept vertically for 60 min to attain adequate extraction. The heptane phase was then carefully transferred into a conical flask using a pipette. The whole process was repeated again to ensure proper extraction. The solvents were then evaporated until visually determined as dry, and the conical flasks were then dried at 105°C for 4 h. The flasks were kept in a desiccator to cool and then weighed. The percentage of total extractable fat was calculated.

### 2.9 Measurement of Overrun

Both frozen dessert mix and frozen dessert sample were weighed in a cup with a fixed volume and overrun was calculated using the following equation:

$$\text{Overrun}\;\left(\%\right)=\frac{\text{Weight of frozen dessert}\;\mathrm{mix}\;\left(g\right)\;–\;\text{Weight of frozen dessert}\;\left(g\right)}{\text{Weight of frozen dessert}\;\left(g\right)}\times 100$$

### 2.10 Determination of Hardness

Hardness of the frozen dessert samples was analyzed by TA‐XT plus texture analyzer (Stable Micro Systems Co. Ltd., Surrey, UK) equipped with a probe (P/2, 2 mm diameter, cylindrical, stainless steel). Frozen dessert samples were shaped in paper cups (3.7 cm top diameter and 3 cm bottom diameter) and stored in the freezer (−18°C) for 2 days. After 2 days, each sample container was quickly removed from the freezer and analysis was completed at room temperature within 1 min to minimize variability due to sample warming. Before the measurements, the paper cups were aligned to allow the probe to penetrate the geometrical center of the samples. The hardness measurement was operated at 2 mm/s pre‐test speed, 2 mm/s test speed, 10 mm/s post‐test speed, 15 mm test distance, and 5 g trigger force. Hardness values (N) were recorded as the peak compression force during penetration.

### 2.11 Measurement of Melting Properties

The melting behavior of the frozen dessert samples was determined according to Goff and Hartel (2013) with modifications. The frozen dessert samples (about 30 g) were placed on a plastic strainer (1 mm × 1 mm opening) and allowed to stand at room temperature (21 ± 1°C). The melted frozen dessert passing through the grid was collected and weighed every 5 min for 60 min. The time when the first drop of the melted frozen dessert fell was recorded and expressed as the first dripping time (min). The melted portion was plotted against time and the slope of the linear curve interval was taken as the melting rate (%/min).

### 2.12 Analysis of Microstructure

The microstructure of melted frozen dessert samples was analyzed using an optical microscope (Olympus BX51, Tokyo, Japan) equipped with a digital camera (Nikon DS‐Filc, Tokyo, Japan). About 0.2 mL of the sample was diluted in 2 mL of distilled water. An aliquot (10 μL) of diluted sample was placed on a glass microscope slide, covered with a coverslip. The samples were observed under the ambient condition at 100× magnification.

### 2.13 Evaluation of Sensory Quality

Sensory evaluation of the frozen dessert samples was performed by 118 participants from students and staffs of Monash University Malaysia. The study protocol was approved by the Monash University Human Research Ethics Committee (Project ID: 30400). Participants evaluated the samples in relation to color and appearance, flavor, and overall acceptability using a 9‐point hedonic scale (1 = dislike extremely to 9 = like extremely), in accordance with ISO recommendations (ISO 2014). A score of 5 was considered the cutoff value for acceptance (Ortakci and Sert 2012). Additionally, body and texture parameters such as coarse, greasy, sandy, and weak were evaluated on another 9‐point scale, with 1 being “not present” to 9 being “very high.” Samples were served in white paper cups, each with 50 mL capacity, and coded with a three‐digit random number. In order to obtain homogeneous conditions, the cups containing frozen desserts were immediately served to the participants after withdrawing from the freezer. Participants were asked to taste a spoonful of the frozen dessert sample and rinse their palate by drinking water before tasting another sample. Participants were also asked about their consumption pattern (never, every day, once in a week, once in a month, less than once in a month) of dairy‐free foods (i.e., dairy‐free milk such as soy/almond milk, dairy‐free ice cream etc.).

### 2.14 Statistical Analysis

All experiments were executed in independent triplicate. The Statistical Package for the Social Sciences (SPSS) version 26.0 (SPSS Inc., Chicago, IL, USA) was used to conduct a one‐way ANOVA test at *p* < 0.05.

## 3 Results and Discussion

### 3.1 Physicochemical Characteristics

Table 2 represents the physicochemical quality attributes of the frozen dessert samples. TS content is a crucial component directly affecting the quality of frozen desserts. Excess TS may result in curded texture, whereas lower contents could cause ice crystal formation and coarse texture (Beegum et al. 2022). Overall, the TS content of the frozen dessert samples varied from 30.04% to 36.97%. Our TS values are comparable to those reported values (28%–38%) of commercial frozen desserts (Goff and Hartel 2013). The frozen dessert prepared from milk protein exhibited higher TS than frozen desserts with brown rice, pea, and soy protein, attributed to the compositional difference of the protein powders used in the frozen dessert preparation.

**TABLE 2:** Physicochemical quality parameters of frozen dessert samples.

	TS (%)	Fat (%)	Protein (%)	TSS (°Brix)	pH	TA (%)
Milk protein	36.97 ± 0.19^a^	10.39 ± 0.16^a^	3.96 ± 0.13^a^	33.24 ± 0.27^a^	6.20 ± 0.08^a^	0.15 ± 0.02^ac^
Soy protein	30.04 ± 0.19^b^	10.37 ± 0.15^a^	4.01 ± 0.15^a^	28.29 ± 0.21^b^	6.44 ± 0.09^b^	0.14 ± 0.02^ab^
Pea protein	30.22 ± 0.12^bc^	10.44 ± 0.13^a^	4.03 ± 0.25^a^	29.93 ± 0.33^c^	6.61 ± 0.12^c^	0.12 ± 0.01^b^
Brown rice protein	30.40 ± 0.22^c^	10.42 ± 0.09^a^	3.91 ± 0.32^a^	30.27 ± 0.82^c^	6.00 ± 0.07^d^	0.17 ± 0.02^c^

*Note:* ^a–d^Values with the different superscript letters within the same column are significantly (*p* < 0.05) different (*n* = 3).

Abbreviations: TA, titratable acidity; TS, total solids; TSS, total soluble solids.

Fats in frozen desserts provide creaminess and body and proper flavor release. Fats also promote sensory perception by lubricating the palate (Goff and Hartel 2013). Concerning the fat content, the frozen dessert samples showed no significant difference as they were added with the same quantity of oil (Table 2).

Proteins are an important component of solids‐non‐fat which generally contribute to the smooth texture along with the body of the frozen desserts through emulsification of the fat, development of foam and supporting the air cells, and escalation of viscosity of the unfrozen phase (Goff and Hartel 2013). A typical frozen dessert should contain at least 2.5% protein. For all frozen dessert formulations, the amount of protein (3.6%) was kept constant (Table 1), and hence, protein content was similar among the frozen desserts (Table 2).

TSS of frozen desserts are generally from sugars, solids‐non‐fat components (i.e., proteins), stabilizers, and emulsifiers (Goff and Hartel 2013). TSS content of frozen desserts ranged from 28.29 to 33.24°Brix, with the frozen desserts containing milk protein showing higher TSS values compared to frozen desserts made using brown rice, pea, and soy protein. These results are in agreement with that of TS values, indicating higher TS in formulation resulted in higher TSS value, similar findings reported by other researchers (Beegum et al. 2022).

With regard to pH and TA, the values varied among the frozen dessert samples (Table 2) and these are in line with the findings of the previous studies (Goff and Hartel 2013; Meneses et al. 2020; Falah et al. 2021; Beegum et al. 2022). The different values of pH and TA are presumably because of the difference in protein types in the formulations.

When comparing frozen desserts containing plant proteins, it was noted that the physicochemical characteristics (i.e., TS, TSS, pH, and TA) varied among brown rice, pea, and soy protein, possibly due to their difference in protein composition.

### 3.2 Color

Color is directly associated with the appearance of a food product and is considered an influential quality attribute that prompts the consumer's acceptability (Meneses et al. 2020). Table 3 depicts the instrumental color parameters of frozen dessert samples. The frozen dessert containing milk protein was whiter and exhibited a higher L* value than frozen desserts prepared with brown rice, pea, and soy protein. The colloidal particles such as milk fat globules and casein micelles might be responsible for the white color that can scatter light in the visible spectrum (Meneses et al. 2020). Values for a* (green to red) and b* (blue to yellow) significantly differed (*p* < 0.05) among frozen dessert samples, which could be because of the difference in composition of the protein powders used.

**TABLE 3:** Color attributes of frozen dessert samples.

	L*	a*	b*	C*	WI
Milk protein	93.09 ± 0.04^a^	−1.64 ± 0.03^a^	16.59 ± 0.16^a^	16.67 ± 0.15^a^	81.96 ± 0.13^a^
Soy protein	91.34 ± 0.04^b^	0.40 ± 0.08^b^	8.28 ± 0.04^b^	8.29 ± 0.04^b^	88.01 ± 0.03^b^
Pea protein	88.39 ± 0.00^c^	1.77 ± 0.11^c^	11.01 ± 0.12^c^	11.15 ± 0.14^c^	83.90 ± 0.10^c^
Brown rice protein	80.88 ± 0.12^d^	4.63 ± 0.04^d^	17.15 ± 0.09^d^	17.76 ± 0.10^d^	73.90 ± 0.15^d^

*Note:* ^a–d^Values with the different superscript letters within the same column are significantly (*p* < 0.05) different (*n* = 3).

Abbreviations: a*, redness; b*, yellowness; C*, chroma; L*, lightness; WI, whiteness index.

C* is the quantitative measure of colorfulness that describes the extent a color differs from a gray color with the same lightness. Greater color intensity of samples will lead to higher C* values (Falah et al. 2021). On the other hand, WI measures consumers' preference for white colors combining lightness and yellow‐blue into a single term (Pathare, Opara, and Al‐Said 2013). Both C* and WI values were significantly different (*p* < 0.05) among frozen dessert samples, presumably due to different proteins addition.

Concerning the color parameters, the frozen desserts with brown rice, pea, and soy protein were observed to be significantly difference from each other, which might be because of the compositional difference of these proteins.

### 3.3 Particle Size Distribution and Fat Destabilization

**FIGURE 1:**
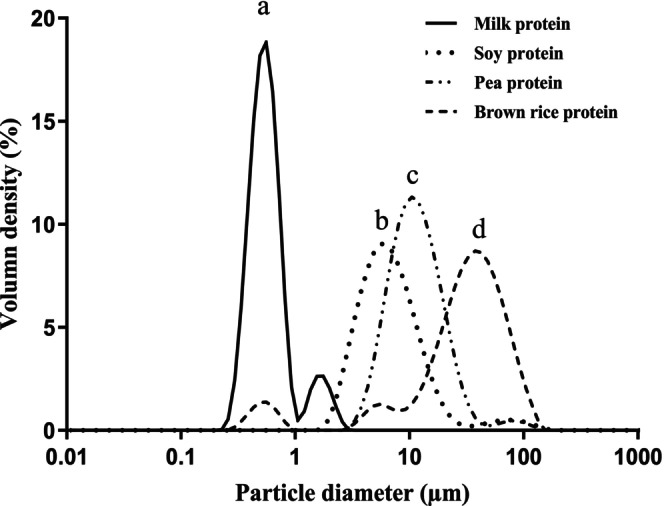
Particle size distribution of frozen dessert samples. ^a–d^Values with the different superscript letters within the same column are significantly (*p* < 0.05) different (*n* = 3).

The average particle diameter (*d*_4,3_) and fat destabilization of frozen dessert samples were evaluated. As noticed in Table 4, the *d*_4,3_ values were in the range of 0.66–36.27 μm. These values were higher compared to frozen dessert mixes, as shown in our earlier study (Hasan, Thoo, and Siow 2023b), suggesting whipping and freezing‐induced fat destabilization (Goff and Hartel 2013). Under the whipping and freezing conditions, the particle size distribution of all frozen desserts dispersed in water was bi‐ or trimodal curves (Figure 1). However, a shift in particle size distribution toward larger size values was noted when compared against frozen dessert mixes. This increase might be due to partial coalescence of the fat globules, since partial coalescence is defined as “an irreversible agglomeration/clustering of fat globules, held together by a combination of fat crystals and liquid fat, and a retention of identity of individual globules as long as the crystal structure is maintained” (Goff 2000). This phenomenon is well‐established and has been observed by others (Cheng, Dudu, Li, et al. 2020; Cheng, Dudu, Wang, et al. 2020; Cheng et al. 2021).

**TABLE 4:** Average particle diameter and partial coalescence degree of frozen dessert samples.

	*d*_4,3_ (μm)	PCD (%)
Milk protein	0.66 ± 0.00^a^	3.09 ± 0.16^a^
Soy protein	12.19 ± 0.37^b^	49.76 ± 1.10^b^
Pea protein	15.14 ± 0.43^c^	64.97 ± 0.91^c^
Brown rice protein	36.27 ± 0.59^d^	111.61 ± 18.37^d^

*Note:* ^a–d^Values with the different superscript letters within the same column are significantly (*p* < 0.05) different (*n* = 3).

Abbreviations: *d*_4,3_, volume weighted average diameter; PCD, partial coalescence degree.

When considering the plant proteins only, the frozen dessert with brown rice protein showed a larger particle size than that of soy and pea protein. This might be due to the lower solubility and emulsifying properties of brown rice protein, as indicated in our earlier study (Hasan, Thoo, and Siow 2023b). It is assumed that poor protein solubility may lead to the large droplet formation because of their inability to migrate to the oil/water interface during emulsification (Karaca, Low, and Nickerson 2011).

Fat droplet destabilization resulting from partial coalescence was evaluated using *d*_4,3_ values obtained from frozen dessert mixes and frozen desserts after dispersion in SDS solution (Table 4). The frozen dessert developed with milk protein exhibited lower PCD value than frozen desserts made using brown rice, pea, and soy protein, indicating milk protein stabilized mixes were more stable against partial coalescence than those stabilized with brown rice, pea, and soy protein. Previous research also reported higher partial coalescence in frozen dessert (i.e., ice cream) with soy protein than that of milk protein (Cheng et al. 2016). This is likely to be due to the steric stabilization of casein micelles in milk proteins leading to the formation of a thicker and more viscoelastic membrane at the oil–water interface. This layer prevents the crystals from piercing the membranes of adjacent fat globules necessary for partial coalescence during whipping and freezing (Fredrick, Walstra, and Dewettinck 2010; Cheng et al. 2021).

Among the dairy‐free options, the frozen dessert with brown rice protein indicated higher levels of fat destabilization than frozen desserts containing soy and pea proteins. According to Fredrick, Walstra, and Dewettinck (2010), a lower protein surface load increases the partial coalescence rate. In our earlier study (Hasan, Thoo, and Siow 2023b), we noticed that the amount of protein adsorbed at the oil/water interface was lower in frozen dessert mix containing brown rice protein than soy and pea protein. Since less protein present, the membrane between oil/water interface becomes very thin and more susceptible to subsequent destabilization (Goff 1997b).

### 3.4 Desorption Index

Desorption refers to phase separation during and after the melting of frozen desserts, indicating a low‐quality product (Meneses et al. 2020). Desorption is considered a relevant quality attribute for frozen desserts, where no desorption is expected. In this study, no frozen dessert formulations presented phase separation (result not shown); hence, desorption was not noticed. It was reported that phase separation after the melting of frozen desserts is greatly influenced by proteins (Meneses et al. 2020). Our results indicate that brown rice, pea, and soy protein have the potential for the production of dairy‐free frozen desserts that are stable to desorption.

### 3.5 Solvent‐Extractable Fat

**FIGURE 2:**
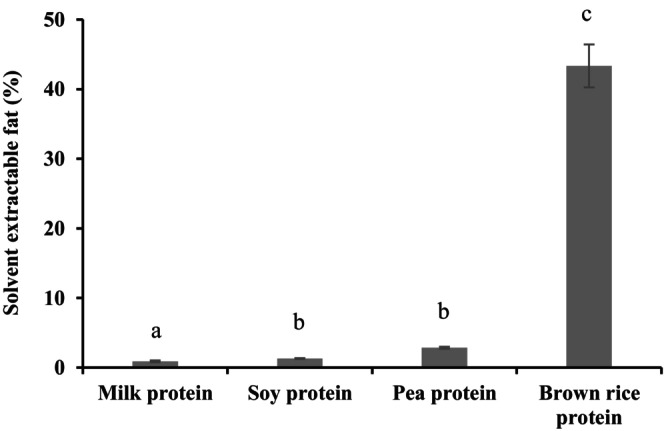
Solvent extractable fat of frozen dessert samples. ^a–c^Values with the different superscript letters within the same column are significantly (*p* < 0.05) different (*n* = 3).

Overall, frozen desserts containing brown rice, pea, and soy protein displayed higher level of solvent‐extractable fat than the frozen desserts made with milk protein (Figure 2). According to Pelan et al. (1997) and Petrut, Danthine, and Blecker (2016), a higher value of solvent‐extractable fat indicates a higher level of fat destabilization by partial coalescence. This implies that a higher quantity of fat underwent partial coalescence when brown rice, pea, and soy protein were present than the presence of milk protein, which coincides with our PCD data (Table 4). It is likely because the casein micelles in milk proteins create a larger steric barrier at the oil/water interface and form a thicker adsorbed layer, impeding partial coalescence to a greater extent than brown rice, pea, and soy protein (Fredrick, Walstra, and Dewettinck 2010). Consequently, a lesser amount of fat is available to interact with the solvent, and thus, a lower solvent extractable fat is observed.

Frozen desserts made with brown rice, pea, and soy protein varied in solvent extractable fat content. The frozen dessert containing brown rice protein showed a greater solvent extractable value than frozen desserts with soy and pea protein (Figure 2), reasonably because of a higher level of destabilized fats that are exposed to the solvent.

### 3.6 Overrun

Overrun is considered one of the essential parameters influencing the textural characteristics of frozen desserts (Goff and Hartel 2013). In the present study, the overrun values of all the frozen dessert samples were between 37.88% and 50.85%, with frozen desserts containing brown rice, pea, and soy protein displaying lower overrun values than the frozen desserts with milk protein (Table 5).

**TABLE 5:** Overrun, hardness, and melting properties of frozen dessert samples.

	Overrun (%)	Hardness (N)	Melting properties	
			First dripping time (min)	Melting rate (%/min)
Milk protein	50.85 ± 1.48^a^	5.34 ± 1.14^a^	16.44 ± 2.07^a^	1.95 ± 0.13^a^
Soy protein	40.78 ± 2.07^b^	22.24 ± 2.25^b^	38.11 ± 2.47^b^	0.19 ± 0.01^b^
Pea protein	37.88 ± 1.10^c^	26.37 ± 2.11^c^	37.33 ± 2.50^b^	0.12 ± 0.01^b^
Brown rice protein	47.50 ± 2.13^d^	20.02 ± 2.35^b^	36.78 ± 2.54^b^	0.29 ± 0.01^c^

*Note:* ^a–d^Values with the different superscript letter within the same column are significantly (*p* < 0.05) different (*n* = 3).

This overrun could be ascribed to several factors. One might be the viscosity of the frozen dessert mixes, which hindered the foaming capacity. Previous research supports this claim that frozen dessert mixes with high viscosities were found to incorporate less air during freezing, and hence, less overrun was noticed (Biasutti et al. 2013; Yuennan, Sajjaanantakul, and Goff 2014). In our earlier work (Hasan, Thoo, and Siow 2023b), we found that frozen dessert mixes with brown rice, pea, and soy protein were more viscous than the frozen dessert mix with milk protein; hence, less overrun was noticed.

Another reason might be the crystalline fat content. According to previous research, increased fat crystals would increase the possibility of rupture of the film between the fat droplet and air bubbles by protruding fat crystals (Kamath et al. 2008; Cheng, Dudu, Li, et al. 2020; Cheng et al. 2021). In our earlier work (Hasan, Thoo, and Siow 2023b), we noted higher crystalline fat (as observed by greater Δ*H*_c_ values) in frozen desserts with brown rice, pea, and soy protein than frozen desserts containing milk protein and, therefore, overrun values decreased.

Higher fat destabilization could also hinder overrun. It has been reported that proteins can adsorb at the air‐serum interface and form a stabilizing film around air bubbles leading to foam formation (Zayas 1997). Fat globules, on the other hand, can act as foam breakers (Kamath et al. 2008). However, partially coalesced fat clumps can attach to the air surfaces and render excellent stabilization to air cells (Méndez‐Velasco and Goff 2011). During whipping and freezing, well‐structured fat aggregates can preferentially displace proteins from the air‐serum interface resulting in reduced foamability (Cheng, Dudu, Li, et al. 2020). It is therefore likely that frozen desserts containing brown rice, pea, and soy protein would reflect lower overrun values than those with milk protein owing to higher fat destabilization.

When the dairy‐free category is considered, the frozen dessert with brown rice protein exhibited higher overrun values than soy and pea protein. This might be due to the fact that the frozen dessert mix containing brown rice protein was less viscous (Hasan, Thoo, and Siow 2023b), and hence, viscosity had less impact on foaming capacity, as discussed above. Additionally, fewer fat crystals in the frozen dessert containing brown rice protein would do less damage to the thin film between the fat droplet and air bubbles and increase overrun.

### 3.7 Hardness

Hardness refers to the resistance of the frozen dessert to deformation when an external force is employed (Muse and Hartel 2004). Table 5 reports the average hardness values of the frozen dessert samples. In general, frozen desserts made with brown rice, pea, and soy protein were harder than those made with milk protein.

A number of factors might be behind this behavior. In our previous study (Hasan, Thoo, and Siow 2023b), we noticed a higher viscosity of frozen dessert mixes with brown rice (119.10 mPa·s), pea (301.10 mPa·s), and soy (247.17 mPa·s) protein compared to the frozen dessert mix with milk protein (74.71 mPa·s). The higher viscosity increased the resistance to penetration by the probe and greater hardness was noticed (Muse and Hartel 2004; Sofjan and Hartel 2004; Liu, Sala, and Scholten 2024).

A new air cell surface is formed during whipping and freezing process, and this air acts as a compressible dispersed phase in the frozen desserts system. When a larger volume of air is entrapped, it contributes to less resistance to the applied force resulting in less hardness of frozen desserts (Muse and Hartel 2004; Sofjan and Hartel 2004; Biasutti et al. 2013; Moolwong, Klinthong, and Chuacharoen 2023; Liu, Sala, and Scholten 2024). Our hardness values support this phenomenon and a lower hardness was noticed with higher overrun values in frozen dessert samples (Table 5).

The level of fat destabilization (Table 4) was also observed to positively influence the hardness of frozen desserts in our study. These results are consistent with those reported by others (Muse and Hartel 2004; Zhao et al. 2023). According to them, the partially coalesced fat globules lead to the formation of a network around the air bubbles and can subsequently increase the hardness of frozen dessert.

Regarding the hardness of plant protein options, the brown rice protein‐made frozen dessert had a lower hardness value than soy and pea protein frozen desserts. This might be due to the lower viscosity of the frozen dessert mix (Hasan, Thoo, and Siow 2023b) and the higher overrun value of the frozen dessert with brown rice protein (Table 5).

### 3.8 Melting Properties

Frozen dessert melting is a combined process concerning both heat and mass transfer (Soukoulis, Chandrinos, and Tzia 2008). Heat gradually transfers into the frozen dessert from the exterior, causing the ice crystals to melt. The newly formed water is diffused into the unfrozen concentrated serum phase, and then the diluted solution flows through the structural elements of frozen dessert and eventually drips (Muse and Hartel 2004; Soukoulis, Chandrinos, and Tzia 2008).

The melting behavior expressed through measuring the first dripping time, and melting rate of frozen desserts are shown in Table 5. According to Table 5, frozen desserts made from brown rice, pea, and soy protein manifested better melting characteristics than the frozen dessert containing milk protein. The first dripping time was significantly (*p* < 0.05) extended in the case of frozen desserts containing brown rice, pea, and soy protein compared to the frozen desserts with milk protein, indicating improved melting resistance. Additionally, the frozen dessert prepared using brown rice, pea, and soy protein had a lower melting rate than the frozen dessert with milk protein.

The melting rate of the frozen dessert samples could reasonably be due to the rheological attributes (viscosity) of the frozen dessert mixes (Hasan, Thoo, and Siow 2023b). The more viscous the mix, the greater resistance to flow that slows the diffusion of water into the concentrated serum phase before its starts flowing from the interior to the exterior of the frozen dessert (Muse and Hartel 2004; Soukoulis, Chandrinos, and Tzia 2008; Liu, Sala, and Scholten 2024).

During whipping and freezing, partially coalesced clumps of fat globules can displace proteins from the air‐serum interface. The destabilized fat aggregates form a three‐dimensional network, which adsorbs and stabilizes on the surface of air bubbles (Muse and Hartel 2004; Méndez‐Velasco and Goff 2011; Zhao et al. 2023; Liu, Sala, and Scholten 2024). These localized fat clumps at the air bubble interface confer resistance to water drainage and retain air bubbles during the meltdown of frozen desserts (Sofjan and Hartel 2004). Hence, sufficient fat globule destabilization can effectively slow the melting rate of frozen desserts as well (Goff and Hartel 2013). Our PCD data (Table 4) are in line with this phenomenon that higher partially coalesced aggregates with brown rice, pea, and soy protein than milk protein might have lowered the meltdown of frozen desserts by effectively holding water and air bubbles together.

Concerning the melting properties of plant proteins, no significant difference was observed in the first dripping time among frozen desserts prepared using brown rice, pea, and soy protein. Regarding the melting rate, brown rice protein frozen dessert melted faster compared to soy pea protein frozen desserts, presumably because of the lower viscosity of the frozen dessert mix (Hasan, Thoo, and Siow 2023b).

### 3.9 Microscopy

**FIGURE 3:**
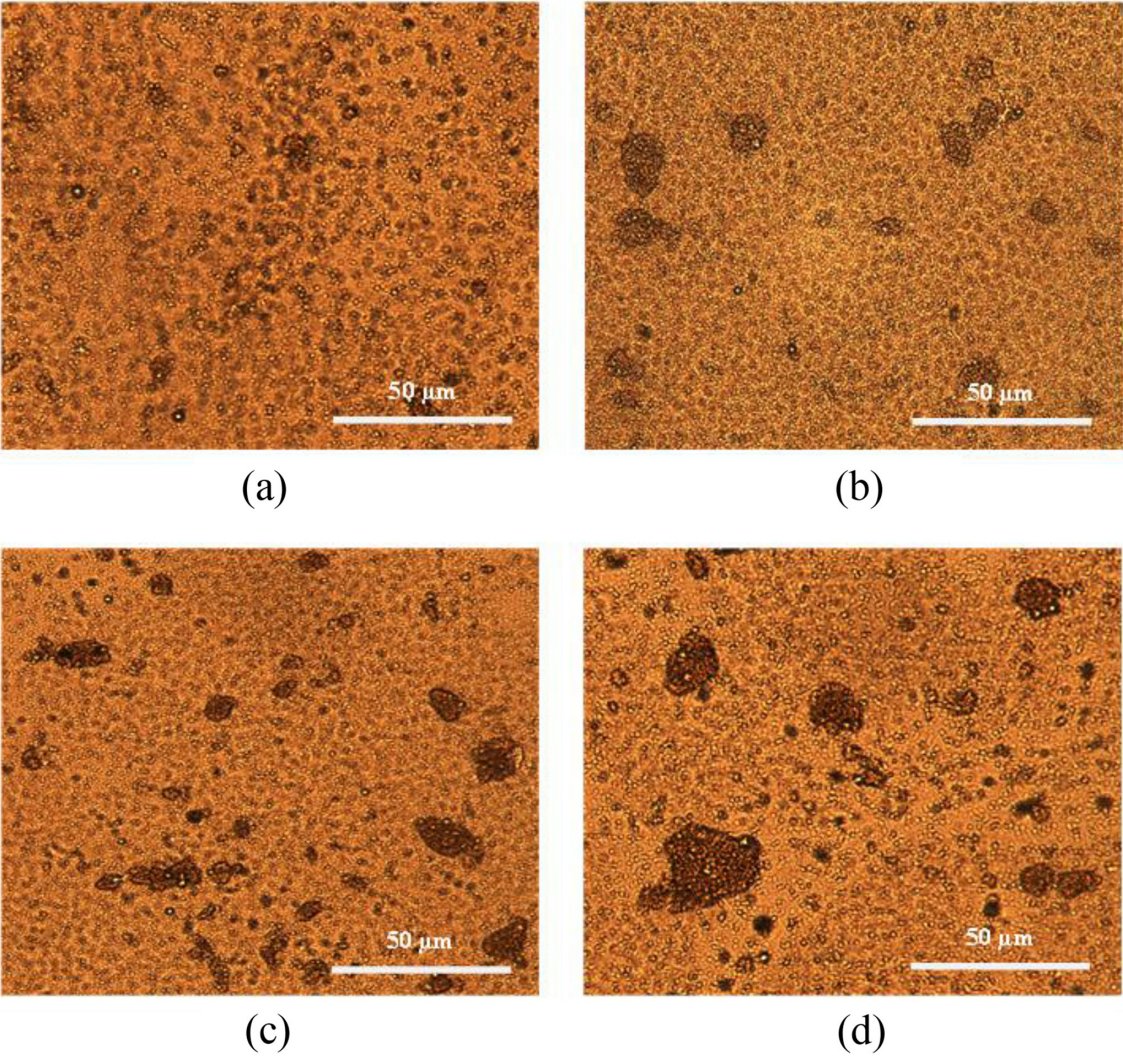
Optical light microscope images (100× magnification) of melted (a) milk protein, (b) soy protein, (c) pea protein, and (d) brown rice protein frozen dessert.

Figure 3 illustrates the optical microscopy images of frozen dessert samples. For the frozen dessert containing milk protein, a few small fat clusters were noticed, whereas most of the fat droplets remained in their individual form (Figure 3a). A different scenario was observed in frozen desserts made from brown rice, pea, and soy protein, where the majority of the fat droplets were presented in clusters, which produced large aggregated particles (Figure 3b–d). These results are in accordance with the particle size and PCD data (Figure 1 and Table 4). A lower fat partial coalescence resulted in fewer fat clusters in the frozen dessert with milk protein, while larger fat clusters formed due to higher partially coalesced fat globules in frozen desserts containing brown rice, pea, and soy protein.

### 3.10 Sensory Analysis

**FIGURE 4:**
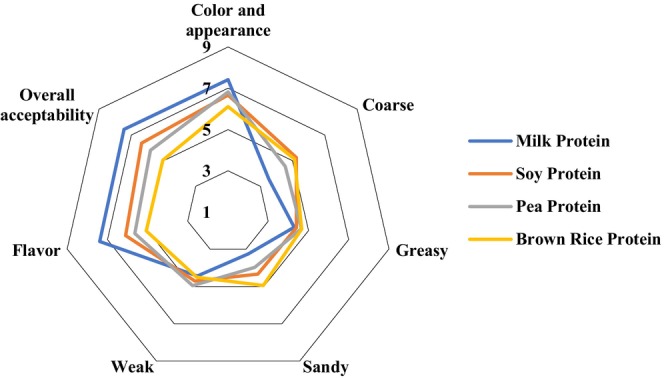
Sensory quality score of frozen dessert samples.

Among the 118 participants, 43.22% were male and 56.78% were female, with age ranging from 20 to 38 years. Most (58.48%) of the participants reported to consume dairy‐free products at least once in a month. The organoleptic evaluation of frozen dessert samples in terms of color and appearance, body and texture, flavor, and overall acceptability is presented in Figure 4. Based on the findings, the sensory perception of frozen dessert samples was significantly influenced by the types of protein used.

Concerning the color and appearance, all frozen dessert samples were given scores above six indicating the liking of color and appearance of the frozen dessert samples. However, frozen desserts containing brown rice, pea, and soy protein received significantly (*p* < 0.05) lower scores for color and appearance than those prepared using milk protein. Our instrumental color analysis supports this finding (Table 3). A lower lightness (L*) value might have resulted in lower color and appearance scores for frozen desserts with brown rice, pea, and soy protein compared to the frozen desserts with milk protein.

Body and textural quality contribute greatly to the acceptance of a food product, including frozen desserts (i.e., ice cream). In this study, a number of textural attributes of the frozen dessert samples, such as coarse, greasy, sandy, and weak, were assessed. For all these parameters, frozen desserts with brown rice, pea, and soy protein had higher scores than those from milk protein; however, participants found no difference (*p* > 0.05) in greasy and weak texture among the samples. It is assumed that higher protein content and fibers of plant protein isolates are responsible for these textural defects (Guler‐Akin, Avkan, and Akin 2021).

The flavor of a product is perceived by the combined sensation of taste and smell, and flavor perception is a vital parameter for accepting a product by consumers. The sense of smell involves various elements, including the detection of volatile compounds released by food when smelled, the ability to perceive soluble substances through taste, and the sensation of bitterness and sourness perceived by nerve stimulation in the mouth and nasal cavity (Guler‐Akin, Avkan, and Akin 2021). Overall, frozen desserts containing brown rice, pea, and soy protein obtained significantly (*p* < 0.05) lower scores regarding flavor than the frozen desserts with milk protein. It could be attributed to the fact that the distinct taste and smell of brown rice, pea, and soy protein might have altered the flavor of the frozen desserts. The participants did not like the flavor, and hence, these samples were scored lower.

In the case of overall acceptability, the participants gave significantly (*p* < 0.05) lower scores for frozen desserts containing brown rice, pea, and soy protein compared to the frozen desserts with milk protein; however, the former ones remained within the acceptable range (≥ 5 out of 9). The characteristic aroma of brown rice, pea, and soy protein might be responsible for this. Plant proteins such as brown rice, pea, and soy protein isolates contain 80% or more protein and are valued greatly in the food industry because of their techno‐functional properties and nutritive values (Guler‐Akin, Avkan, and Akin 2021). However, during extraction and processing, these proteins undergo several transformations resulting in a degradation of the desirable endogenous fragrance active compounds. Several studies have also reported a lower acceptability of frozen desserts using plant proteins (Pereira et al. 2011; Ahanian, Pourahmad, and Mirahmadi 2014; Guler‐Akin, Avkan, and Akin 2021).

When comparing sensory quality within the dairy‐free category, it was noted that the frozen dessert containing brown rice protein received a lower sensory score in terms of color, flavor, and overall acceptability compared to frozen desserts containing soy and pea protein. However, no significant difference was observed among frozen desserts containing brown rice, pea, and soy protein concerning the body and texture parameters (coarse, greasy, sandy, and weak). Hence, the dairy‐free frozen desserts can be ordered as soy protein > pea protein > brown rice protein based on the sensory acceptance score; however, all frozen desserts remained acceptable.

## 4 Conclusion

The findings of the present study demonstrated that the source of protein significantly influenced the physicochemical properties and sensory acceptance of frozen dessert samples. Frozen desserts containing brown rice, pea, and soy protein showed greater resistance to melting, but they scored lower in sensory quality than the frozen desserts with milk protein; nevertheless, they remained at an acceptable level. When compared among the plant proteins, the physicochemical characteristics varied within frozen desserts containing brown rice, pea, and soy protein because of their difference in protein compositions. The frozen dessert with brown rice protein showed higher overrun, lower hardness, and higher melting rate than frozen desserts containing soy and pea protein. Additionally, the brown rice protein‐made frozen dessert received a lower sensory acceptance than soy and pea protein frozen desserts. Among the dairy‐free category, the frozen desserts can be ordered as soy protein > pea protein > brown rice protein based on overall analyses. Hence, it can be concluded that brown rice, pea, and soy proteins showed potential to be used as viable alternatives to milk protein for the development of dairy‐free frozen dessert. The current findings will also provide useful guidance for the food industries to enhance the properties of frozen desserts (such as ice cream) without using dairy ingredients. It should be noted that we observed relatively lower sensory quality of dairy‐free frozen desserts than their dairy counterpart and plant proteins (i.e., soy, pea, and brown rice protein) are mostly attributed to this. Therefore, future research could be focused to mask or remove the beany flavor of plant proteins (i.e., extrusion and 3D printing in addition to extraction, purification, and functionalization) to improve the sensory acceptance of food products containing plant‐based proteins, including ice cream. Moreover, further tests should be conducted before commercialization of the product (i.e., stability, shelf life, etc.).

## Author Contributions

**Towhid Hasan:** conceptualization (lead), formal analysis (lead), methodology (lead), writing – original draft (lead). **Yin Yin Thoo:** resources (supporting), supervision (supporting), writing – review and editing (equal). **Lee Fong Siow:** conceptualization (supporting), funding acquisition (lead), resources (lead), supervision (lead), writing – review and editing (equal).

## Conflicts of Interest

The authors declare no conflicts of interest.

## Data Availability

The data that support the findings of this study are available in the manuscript.
